# Quantitative assessment of intra‐ and inter‐modality deformable image registration of the heart, left ventricle, and thoracic aorta on longitudinal 4D‐CT and MR images

**DOI:** 10.1002/acm2.13500

**Published:** 2021-12-27

**Authors:** Alireza Omidi, Elisabeth Weiss, John S. Wilson, Mihaela Rosu‐Bubulac

**Affiliations:** ^1^ Department of Biomedical Engineering, College of Engineering Virginia Commonwealth University Richmond Virginia USA; ^2^ Department of Radiation Oncology Virginia Commonwealth University Health Richmond Virginia USA; ^3^ Pauley Heart Center Virginia Commonwealth University Health System Richmond Virginia USA

**Keywords:** cardiotoxicity, deformable image registration, multimodality

## Abstract

**Purpose:**

Magnetic resonance imaging (MRI)‐based investigations into radiotherapy (RT)‐induced cardiotoxicity require reliable registrations of magnetic resonance (MR) imaging to planning computed tomography (CT) for correlation to regional dose. In this study, the accuracy of intra‐ and inter‐modality deformable image registration (DIR) of longitudinal four‐dimensional CT (4D‐CT) and MR images were evaluated for heart, left ventricle (LV), and thoracic aorta (TA).

**Methods and materials:**

Non‐cardiac‐gated 4D‐CT and T1 volumetric interpolated breath‐hold examination (T1‐VIBE) MRI datasets from five lung cancer patients were obtained at two breathing phases (inspiration/expiration) and two time points (before treatment and 5 weeks after initiating RT). Heart, LV, and TA were manually contoured. Each organ underwent three intramodal DIRs ((A) CT modality over time, (B) MR modality over time, and (C) MR contrast effect at the same time) and two intermodal DIRs ((D) CT/MR multimodality at same time and (E) CT/MR multimodality over time). Hausdorff distance (HD), mean distance to agreement (MDA), and Dice were evaluated and assessed for compliance with American Association of Physicists in Medicine (AAPM) Task Group (TG)‐132 recommendations.

**Results:**

Mean values of HD, MDA, and Dice under all registration scenarios for each region of interest ranged between 8.7 and 16.8 mm, 1.0 and 2.6 mm, and 0.85 and 0.95, respectively, and were within the TG‐132 recommended range (MDA < 3 mm, Dice > 0.8). Intramodal DIR showed slightly better results compared to intermodal DIR. Heart and TA demonstrated higher registration accuracy compared to LV for all scenarios except for HD and Dice values in Group A. Significant differences for each metric and tissue of interest were noted between Groups B and D and between Groups B and E. MDA and Dice significantly differed between LV and heart in all registrations except for MDA in Group E.

**Conclusions:**

DIR of the heart, LV, and TA between non‐cardiac‐gated longitudinal 4D‐CT and MRI across two modalities, breathing phases, and pre/post‐contrast is acceptably accurate per AAPM TG‐132 guidelines. This study paves the way for future evaluation of RT‐induced cardiotoxicity and its related factors using multimodality DIR.

## 1 INTRODUCTION

Deformable image registration (DIR) is an essential tool in radiotherapy (RT) applications.^1^ This process maps one image set onto another image set using nonlinear transformations to register the anatomical differences between the two images locally, rather than using a rigid transformation that only undergoes translations and rotations.^2^, ^3^ For both clinical practice and research, an increasing amount of multimodal and longitudinal imaging data before, during, and after RT treatment has led to an increased need for reliable DIR.^2^ Current applications of DIR in RT include mono‐ and multimodal image fusion for motion‐tracking and image segmentation, treatment adaptation, and monitoring of the treatment response in terms of therapeutic efficacy and secondary toxicity.^2^, ^4^, ^5^, ^6^, ^7^

One specific need for DIR is to correlate quantitative RT dosimetry maps from planning computed tomographies (CTs) to longitudinal imaging data from a structure of interest to assess the time‐course and severity of changes due to RT‐induced cardiovascular toxicity (CVT).^8^, ^9^ Notably, CVT is found to be a competing risk factor for survival in cancer patients who receive thoracic RT,^10^ and cardiovascular magnetic resonance (MR) may provide early indicators of CVT which could assist in initiating preventive measures to mitigate progression of myocardial damage.^11^ For example, Umezawa et al.^12^ recently demonstrated dose‐dependent cardiotoxicity for myocardium receiving more than 30 Gy by using late gadolinium enhancement (LGE) MR that utilized rigid registration between planning CT and pre‐treatment MR in addition to deformable registration between pre‐ and post‐treatment MR to monitor changes before and after RT treatment. Similarly, a cross‐sectional study by Ricco et al.^13^ assessed the relation between RT and myocardial fibrosis using rigidly registered CT planning and MR‐derived post‐treatment LGE and T1‐mapping data. In addition, image registration is also debuting in non‐oncologic radiation medicine, with radioablation for the treatment of cardiac arrhythmias such as ventricular tachycardia and atrial fibrillation being an emerging treatment method with a promising outlook.^14^

Regardless of the clinical purpose, robust DIR is critical for applications in the lung, heart, and mediastinum due to the large motion of thoracic organs during the cardiac and respiratory cycles to account for variations in the position of locations of interest and multimodal differences in cardiac and respiratory gating approaches. Notably, in many clinical settings, cardiac and respiratory gating are not performed equally for all imaging modalities used for RT. For example, planning CTs may be ungated, respiratory‐gated but not cardiac‐gated, cardiac‐gated but only respiratory‐gated at a single phase of the respiratory cycle (e.g., during a breath‐hold), or other unique combination of gating techniques.^15^ However, diagnostic MR imaging, due to its longer acquisition time, may be respiratory‐gated for imaging of the lung, but not cardiac‐gated unless the pathology directly involves the heart. Thus, in addition to the classic difficulty in multimodal DIR, these differences in cardiorespiratory gating can make DIR not only challenging, but specific for particular pathologies and/or institutions (due to available technology and departmental procedure). As a result, planning for any large study of RT efficacy and/or secondary toxicity that requires DIR of images across modalities or time for spatiotemporal correlations may first require an assessment of the reliability of the planned DIR for the specific imaging techniques and target organs.

The goal of this study, therefore, is to conduct a quantitative intra‐ and intermodal DIR assessment for CT and MR imaging conducted before and during RT for a cohort of patients with lung cancer focusing on three regions of interest to be used for future assessment of RT‐induced CVT: the whole heart, the left ventricle (LV), and the thoracic aorta (TA). Notably, the whole heart and/or LA have been the most common structures of interest to evaluate correlations between radiation dose and post‐RT cardiac toxicity (i.e., dysfunction). Herein, we also include the TA as fewer studies have considered aortic toxicity as a potential source of primary dysfunction (and secondary source of inducing/worsening cardiac function). Specific imaging includes paired longitudinal non‐cardiac‐gated CT and MR imaging at various breathing phases and contrast levels. While the results for this study are specific for the imaging involved, the quantitative DIR assessment presented herein may provide a useful framework for evaluating future DIR applications using different imaging techniques or gating parameters.

## 2 METHODS AND MATERIALS

### 2.1 Data acquisition

Following IRB approval and informed consent, multimodality imaging of five patients undergoing RT for non‐small cell lung cancer (NSCLC) acquired before treatment and at 5 weeks after initiating treatment was retrospectively analyzed. For each patient, non‐cardiac‐gated free‐breathing four‐dimensional computed tomography (4D‐CT) and T1 volumetric interpolated breath‐hold examination (VIBE) pre‐ and post‐contrast MR images in both inspiration and expiration were obtained. A few patients did not have full datasets: one patient lacked pre‐contrast MR at expiration and two patients lacked pre‐contrast MR at inspiration. CT data at the 0% and 50% respiratory cycle were selected as inspiration and expiration, respectively. Table 1 summarizes the data available for each scenario. 4D‐CT scans were obtained on a Brilliance Big Bore scanner (Philips Healthcare, Cambridge, MA, USA) using the following parameters: 397 mAs, 120 kVp, and 3 mm thickness. T1 VIBE MRI scans were performed with a 1.5T Avanto MRI System (Siemens, Germany) with the following parameters:nTime to Echo (TE) 1.28 ms, Repetition Time (TR) 3.56 ms, Echo Train Length (ETL) 1, slice thickness 2 mm, flip angle 12°, contrast agent 18 ml gadopentetate dimeglumine.

**TABLE 1:** Summary of available imaging data (number of scans) and its use for each scenario investigated

Image sets	Timepoint 1	Timepoint 2	Total
0% (inhale) form 4D‐CT	3 (used in Groups A, D, E)	3 (used in Groups A, D, E)	6
50% (exhale) form 4D‐CT	5 (used in Groups A, D, E)	5 (used in Groups A, D, E)	10
MR – Exhale voluntary breath‐hold no contrast	4 (used in Groups B, C, D, E)	4 (used in Groups B, C, D, E)	8
MR – Inhale voluntary breath‐hold no contrast	3 (used in Groups B, D, E)	3 (used in Groups B, D, E)	6
MR – Exhale voluntary breath‐hold with contrast	5 (used in Group C)	5 (used in Group C)	10

Scenario	CT	MR	CT	MR	Total
CT modality over time (Group A)	8	–	8	–	16
MR modality over time (Group B)	–	7	–	7	14
MR modality contrast effect (Group C)	–	8	–	8	16
CT/MR multimodality at the same time (Group D)	7	7	7	7	28
CT/MR multimodality over time (Group E)	7	–	–	7	14

*Note*: Timepoint 1: pre‐treatment, Timepoint 2: 5 weeks after initiating radiotherapy (RT).

Abbreviations: CT, computed tomography; MR, magnetic resonance; 4D‐CT, four‐dimensional CT.

### 2.2 Contouring

On each dataset, the whole heart, LV, and TA were manually contoured based on contouring guidelines^16^, ^17^, ^18^ and anatomic landmarks using MIM software (MIM Software Inc., Cleveland, OH, USA). Whole heart was contoured from the bifurcation of the right and left pulmonary arteries to the cardiac apex. The TA was contoured from the aortic valve, through the arch, and down the descending TA to the level of the cardiac apex. The LV was contoured from the level of the mitral and aortic valves to the apex, including the septum and free wall. The percent relative volume difference (RVD) for each area of interest was compared between various registration scenarios to check volumetric consistency. All segmentations were reviewed by an experienced radiation oncologist.

### 2.3 Registrations

**FIGURE 1:**
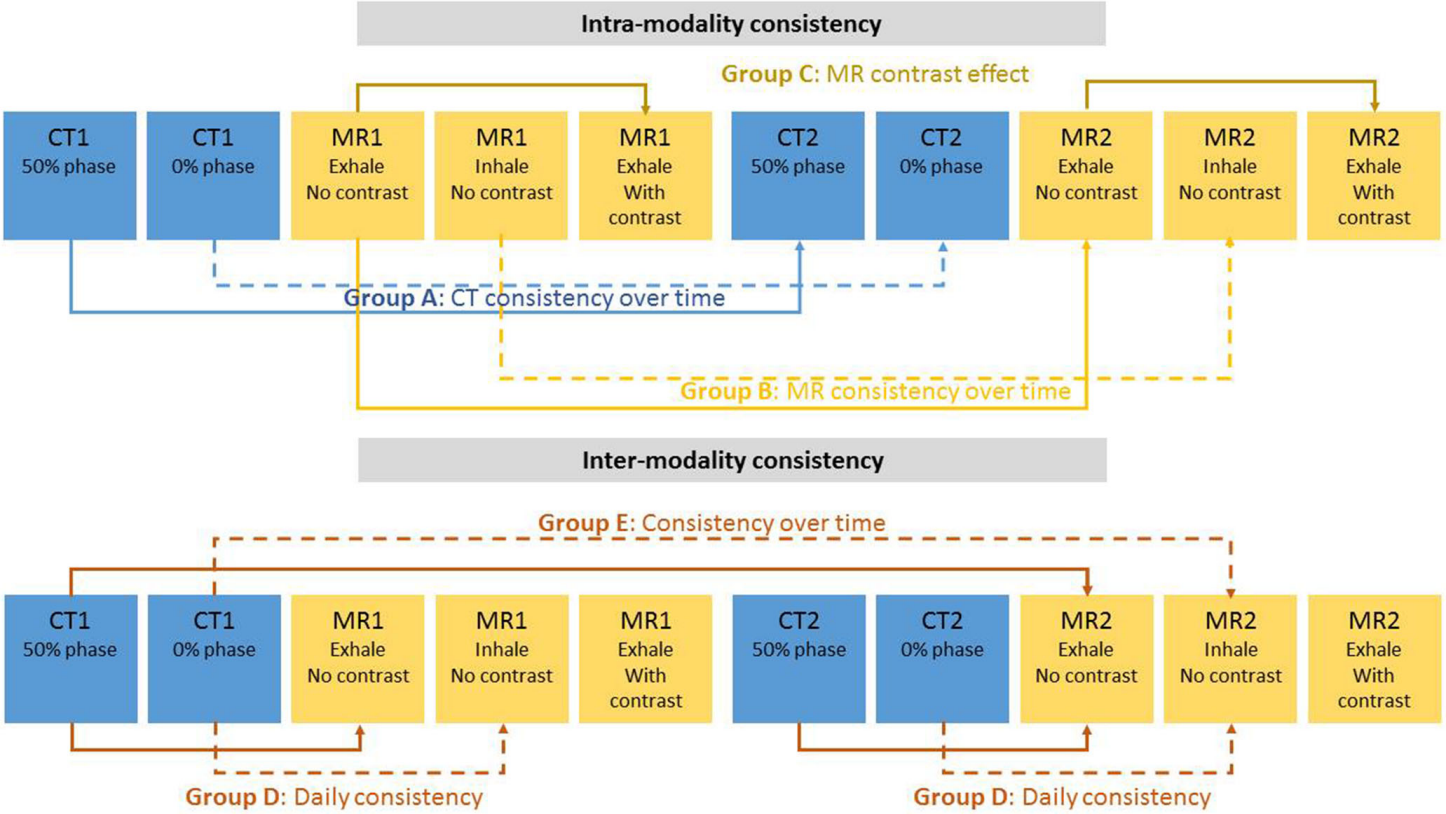
Summary of deformable image registration (DIR) scenarios

Five unique DIRs were performed (three intramodal, two intermodal), as summarized in Figure 1. Intramodal DIRs consisted of: Group A – CT modality over time (same phase of breathing cycle at baseline vs. 5 weeks during RT), Group B – MR modality over time (same phase of breathing cycle at baseline vs. 5 weeks during RT, pre‐contrast), and Group C – MR contrast effect (pre‐ and post‐contrast MR at the same phase of breathing cycle taken at the same time point). Intermodal DIR consisted of: Group D – CT/MR multimodality at the same time (CT and pre‐contrast MR at the same phase of the breathing cycle taken at the same time point) and Group E – CT/MR multimodality over time (CT at baseline vs. pre‐contrast MR at 5 weeks during RT at the same phase of the breathing cycle). For all DIRs involving CT, the earliest CT images were considered as the primary dataset for registration, and the other imaging data were defined as the secondary dataset. For DIRs involving only MR, the earliest pre‐contrast MR images were selected as the primary datasets, and the other imaging data were defined as the secondary dataset.

To conduct the DIR, the primary and secondary datasets were initially fused by rigid image registration (RIR), with the registration narrowed to the thorax to include only heart, LV, and TA. Each RIR process was manually refined, and a subsequent automated RIR was performed, if needed, until the registration result was deemed acceptable by visual inspection by the user. It is recognized in the literature that in the absence of the ground truth, any deformation metrics should always be complemented by an expert visual inspection and human deliberation.^19^ Next, the images underwent DIR encompassing the same area as the RIR. The DIR algorithm used in MIM software is an intensity‐based free‐form algorithm to account for both large deformations and local differences. In order to find the point‐by‐point corresponding locations between the primary and secondary datasets, a grid of control points is detected on the primary dataset using a coarse‐to‐fine multi‐resolution approach. The algorithm begins by accounting for gross differences using a coarse grid. The resolution is then increased and local changes are addressed over a small scale. Optimization is applied by a gradient descent‐based approach and the quality of match is evaluated by an intensity‐based sum of square differences strategy.^20^, ^21^ Finally, the contours of the deformed secondary dataset were transferred to the primary dataset for analysis. All registration processes were performed on the MIM software.

### 2.4 Analysis

The contours of the primary dataset and the transferred deformed secondary dataset were compared using Hausdorff distance (HD), mean distance to agreement (MDA), and Dice metrics. HD is defined as the largest distance of point‐by‐point comparisons from each point in one dataset set to the nearest point in the other dataset set. As a result, it is sensitive to outliers.^19^ MDA is the mean distance of the point‐by‐point comparison approach used in defining the HD and is introduced to alleviate the sensitivity to outliers.^19^, ^22^ Dice is a spatial overlap index, with 0 indicating no overlap and 1 indicating complete overlap.^19^ All quantifications of registration accuracy were performed using the MIM software.

### 2.5 Statistics

Each available dataset (e.g., HD value of the heart for Group A in each patient) was considered an independent sample for the corresponding statistical analysis. Statistical comparisons of mean values between heart, LV, and aorta under each registration scenario for each metric (HD, MDA, and Dice) were performed using Analysis of Variance (ANOVA) with post hoc Tukey's test, as appropriate for the given variance assessed by visual inspection or using the Brown–Forsythe test. t‐tests were used to compare means of each metric for the same area of interest between different scenarios as follows: (1) Group A versus Group B, (2) Group A versus Group C, (3) Group A versus Group D, (4) Group A versus Group E, (5) Group B versus Group C, (6) Group B versus Group D, (7) Group B versus Group E, and (8) Group D versus Group E. Finally, the correlation of average volumes of each structure (heart, LV, TA) between CT and MR data was calculated using Pearson correlation tests. All statistical analyses were performed using JMP software (version Pro 14, SAS Institute Inc., NC, USA), with significance defined as *p* < 0.05.

## 3 RESULTS

**FIGURE 2:**
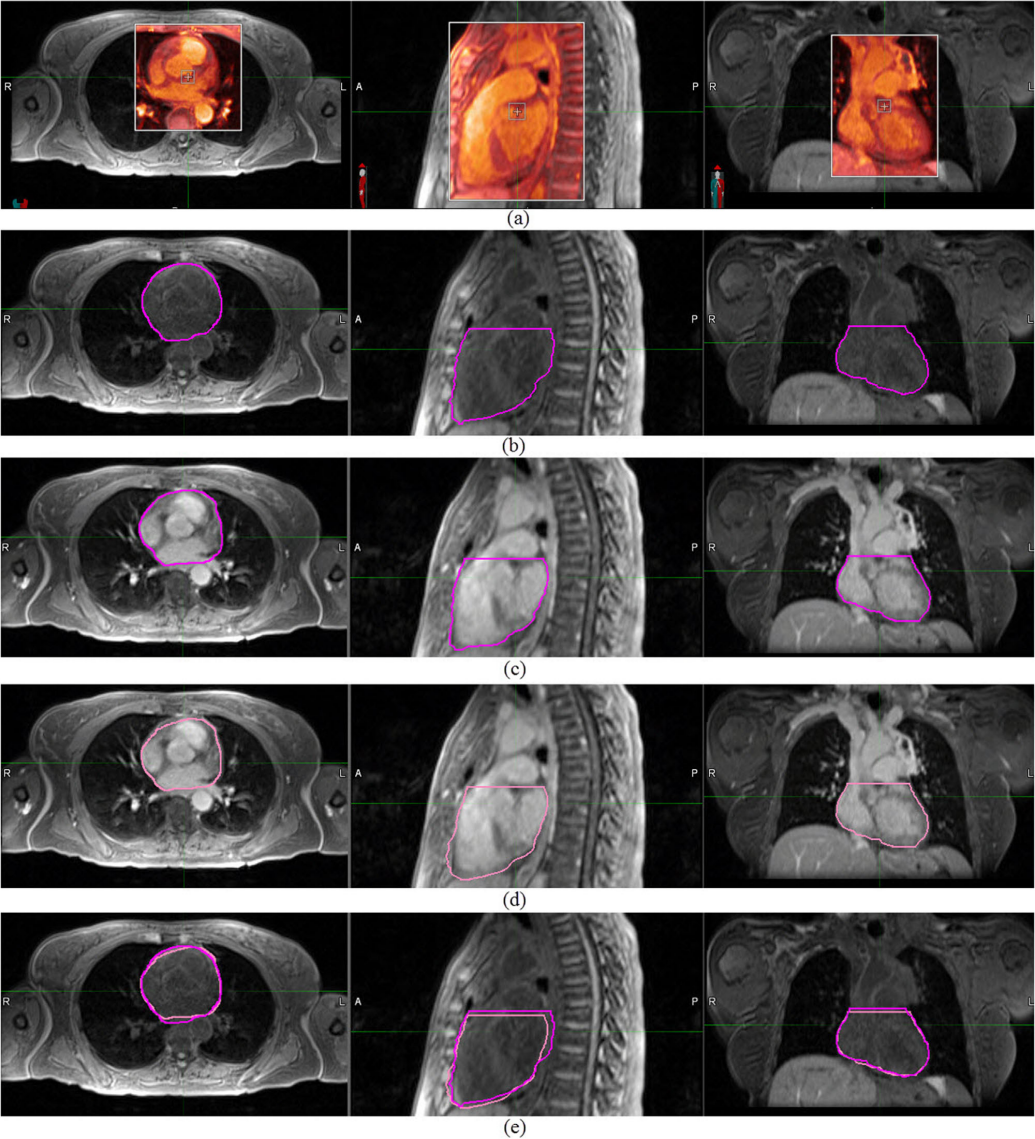
Representation of deformable image registration (DIR) process for magnetic resonance (MR) modality contrast effect scenario (Group C) for the whole heart contour. (a) MR modality contrast effect DIR at expiration, (b) primary contour, (c) secondary contour, (d) deformed secondary contour, and (e) overlap of the deformed secondary contour over the primary one

**FIGURE 3:**
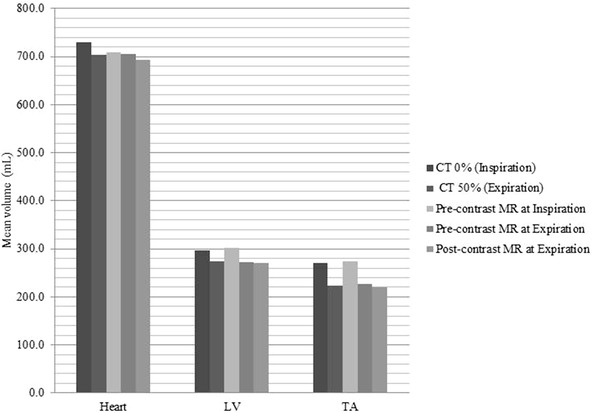
Mean undeformed volumes of the heart, left ventricle (LV), and thoracic aorta (TA) (before deformable image registration (DIR)) under various modalities, breathing phases, and contrasts

An example of the DIR process for pre‐ and post‐contrast MR data at expiration is shown in Figure 2 and displays the fused image, the primary contour, secondary contour, deformed secondary contour, and overlap of the primary and deformed secondary contours. Mean undeformed volumes of the heart, LV, and TA (before DIR) for each modality, breathing phase, and contrast are shown in Figure 3. Comparisons of individual segmented volumes before DIR in all subjects and regions of interest at the same time points (Groups C and D) produced RVD < 5%. Comparisons of individual aortic volumes from imaging at different time points (Groups A, B, and E) produced RVD < 5% in all datasets. For heart and LV, RVD was <10% in all datasets and <5% in approximately half of the datasets. Mean RVD (±SD) values (before the registration process and any deformations) for each registration and region of interest are recorded in Table 2. All mean RVD were <2%. Correlations between volumes from CT and MR data for all regions of interest under various breathing phases and contrast levels were high (*r* = 0.99, *p* < 0.0001).

**TABLE 2:** Percent relative volume difference (RVD) mean (±SD) of the heart, left ventricle (LV), and thoracic aorta (TA) (before registration process and any deformations) under various registration scenarios

	Group A	Group B	Group C	Group D	Group E
Heart (%RVD, mean ± SD)	−1.9 ± 3.8	−0.1 ± 4.3	0.7 ± 0.8	−1.7 ± 2.0	−1.6 ± 4.7
LV (%RVD, mean ± SD)	−0.6 ± 3.1	0.3 ± 3.6	−0.3 ± 2.0	−0.5 ± 2.9	−0.4 ± 4.2
TA (%RVD, mean ± SD)	0.1 ± 2.5	−1.0 ± 2.9	0.6 ± 2.7	0.0 ± 2.2	0.8 ± 2.5

**FIGURE 4:**
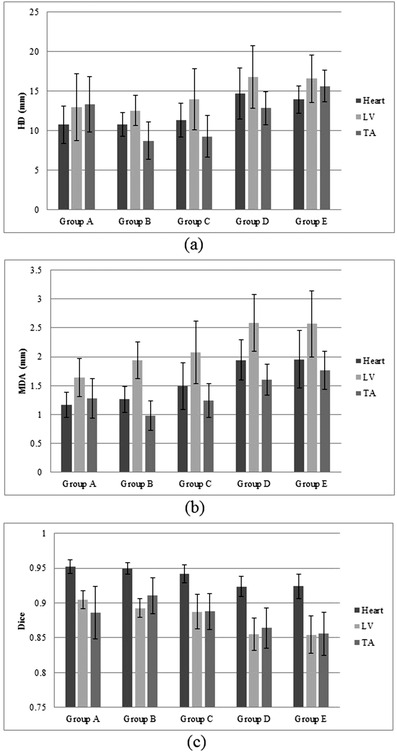
(a) Mean distance to agreement (MDA) (±SD), (b) Hausdorff distance (HD) (±SD), and (c) Dice (±SD) values of different deformable image registration (DIR) scenarios at heart, left ventricle (LV), and thoracic aorta (TA). Group A: computed tomography (CT) modality over time, Group B: magnetic resonance (MR) modality over time, Group C: MR modality contrast effect at the same time, Group D: CT/MR multimodality at the same time, and Group E: CT/MR multimodality over time

Mean DIR metrics (±SD) for each region of interest and registration scenario are graphically shown in Figure 4. For the intramodal registrations, Group A (CT modality over time) demonstrated population means (min–max) of HD, MDA, and Dice values between 10.7 and 13.3 mm (7.3–20.0 mm), 1.1 and 1.6 mm (0.9–2.1 mm), and 0.88 and 0.95 (0.83–0.96), respectively, when considering all regions of interest. For Group B (MR modality over time), population means (min–max) of HD, MDA, and Dice were between 8.7 and 12.5 mm (5.2–16.5 mm), 0.9 and 1.9 mm (0.6–2.3 mm), and 0.89 and 0.95 (0.86–0.95), respectively. Group C (MR modality contrast effect) means (min–max) were between 9.2 and 13.9 mm (5.0–23.3 mm), 1.2 and 2.0 mm (0.9–2.9 mm), and 0.88 and 0.94 (0.84–0.96), respectively. For the intermodal registrations, Group D (CT/MR multimodality at same time) means (min–max) of HD, MDA, and Dice were between 12.8 and 16.8 mm (9.9–24.3 mm), 1.6 and 2.5 mm (1.1–3.7 mm), and 0.85 and 0.92 (0.80–0.94), respectively, and Group E (CT/MR multimodality overtime) means (min–max) were between 13.9 and 16.5 mm (10.5–22.5 mm), 1.7 and 2.5 mm (1.3–3.8 mm), and 0.85 and 0.92 (0.80–0.94).

Results of statistical analyses between registration scenarios are reported in Table 3. Notably, almost all significant differences between scenarios were found comparing registrations between Group A or B and those from Group D or E (i.e., comparisons of intra‐ to intermodal registrations). Specifically, whole heart and LV registrations were significantly worse in intermodal registrations (Groups D and E) in all metrics compared to either intramodal CT or intramodal MR registrations (Groups A and B), except for HD comparison of LV for intramodal CT. Intramodal MR aortic registrations (Group B) were significantly better than intermodal registrations (Groups D and E) for all metrics (except for MDA in Group E), but were only significantly better than intramodal CT registration (Group A) for HD.

**TABLE 3:** *T*‐test results (*p*‐values) comparing mean Hausdorff distance (HD), mean distance to agreement (MDA), and Dice metrics between registrations for each region of interest

*T*‐test		Group B			Group C			Group D			Group E		
		HD	MDA	Dice	HD	MDA	Dice	HD	MDA	Dice	HD	MDA	Dice
Group A	Heart	0.97	0.44	0.56	0.86	0.27	0.39	**0.009**	**0.021**	**0.0001**	**0.01**	**0.007**	**0.007**
	LV	0.81	0.11	0.13	0.78	0.15	0.14	0.07	**0.0002**	**0.0001**	0.1	**0.006**	**0.003**
	TA	**0.016**	0.08	0.2	0.14	0.63	0.58	0.7	0.07	0.15	0.15	**0.024**	0.13
Group B	Heart	–	–	–	0.86	0.53	0.63	**0.009**	**0.033**	**0.0005**	**0.006**	**0.013**	**0.008**
	LV	–	–	–	0.43	0.7	0.8	**0.018**	**0.0068**	**0.0013**	**0.019**	**0.042**	**0.013**
	TA	–	–	–	0.99	0.28	0.41	**0.001**	**0.0001**	**0.002**	**0.0001**	**0.0007**	**0.0068**
Group D	Heart	–	–	–	–	–	–	–	–	–	0.57	0.96	0.96
	LV	–	–	–	–	–	–	–	–	–	0.88	0.93	0.97
	TA	–	–	–	–	–	–	–	–	–	**0.01**	0.27	0.57

Abbreviations: LV, left ventricle; TA, thoracic aorta.

Bold values are significance *p* <0.0001.

Results of comparisons between regions of interest for each registration and metric are reported in Table 4. Overall, whole heart registration was significantly better than LV and aortic registration by Dice in all groups and was significantly better than LV by MDA in all groups but Group E. Though there was no difference in Dice, the TA demonstrated significantly better registration than the LV by HD and MDA in Groups B, C, D, and E (all MR‐based comparisons), except HD in Group E.

**TABLE 4:** Results (*p*‐values) following ANOVA and post hoc Tukey test comparing regions of interest for each metric and registration

		HD	MDA	Dice
Group A	LV‐heart	0.46	**0.018**	**0.0009**
	LV‐TA	0.98	0.082	0.31
	Heart‐TA	0.36	0.63	**0.001**
Group B	LV‐heart	0.125	**0.004**	**0.002**
	LV‐TA	**0.02**	**0.002**	0.37
	Heart‐TA	0.096	**0.04**	**0.01**
Group C	LV‐heart	0.24	**0.03**	**0.0009**
	LV‐TA	**0.02**	**0.003**	0.99
	Heart‐TA	0.42	0.27	**0.001**
Group D	LV‐heart	0.093	**0.0003**	**0.0001**
	LV‐TA	**0.005**	**0.0001**	0.57
	Heart‐TA	0.18	0.069	**0.0001**
Group E	LV‐heart	0.2	0.07	**0.002**
	LV‐TA	0.6	**0.01**	0.2
	Heart‐TA	0.2	0.52	0.7

Abbreviations: HD, Hausdorff distance; LV, left ventricle; MDA, mean distance to agreement; TA, thoracic aorta.

Bold values are significance *p* <0.0001.

## 4 DISCUSSION

American Association of Physicists in Medicine (AAPM) Task Group (TG)‐132 recommendations for acceptable DIR error require MDA <3 mm and Dice >0.8.^1^ For all applications in this study, mean MDA <2.6 mm, mean HD <16.8 mm, and mean Dice >0.85, confirming acceptable registration accuracy. These values are similar to previous studies of CT‐based image registration for tumor and neighboring thoracic tissues, including DIR validation and automatic contour propagation in 4D‐CT lung RT planning, which reported values of MDA ∼2–3 mm, HD ∼20–25 mm, and Dice ∼0.8–0.85.^22^, ^23^ In other studies, Dice coefficient ranged from 0.73 ± 0.08 to 0.95 ± 0.04 for auto‐segmentation of cardiac substructures in averaged 4D‐CT and 0.91–0.93 for multiatlas‐based auto‐segmentation of the heart and all cardiac chambers using multicenter and multivendor computer tomography angiography (CTA).^24^, ^25^ When considering each individual result in this study, every MDA and Dice measurement under each population and scenario met the AAPM guidelines except for three out of 132 measurements (∼2% of the population). The three exceptions were for LV MDA for intermodal DIRs (two in Group D and one in Group E; MDA for these three ranged from 3.3 to 3.8 mm).

For the whole heart and LV, comparisons of results between intramodal (Group A or B) and intermodal DIR (Group D or E) suggest greater accuracy when registering within the same modality (either by CT or MR), even though the intramodal registrations were across different time points. This may be due to dissimilarities in intensity mapping and structural characteristics between different imaging modalities.^26^, ^27^ For the TA, only the MR intramodal registration (Group B) was significantly better than the intermodal CT/MR registrations (Groups D and E). This may partially be due to the TA registration being significantly better than the LV in some metrics in Groups D and E and/or to the TA being better registered intramodally by MR than CT. The latter possibility is partially supported by all metrics being worse on average when registering the TA by CT rather than MR (Group A vs. Group B, Figure 4), though only the HD metric reached significance. Further studies with larger datasets will be required for follow‐up.

The statistically worse registration overall of the LV compared to the whole heart (MDA and Dice) and aorta (MDA) likely relates to the greater volumetric deformation of the LV during the cardiac cycle (normal LV ejection fraction >55%) compared to the entire heart (∼8%)^28^ and TA (∼2%–11%, depending on age).^29^ Due to the lack of cardiac gating in these scans, increased deformation could lead to increased errors in registration. Difficulties in accurately segmenting the LV boundary, particularly in non‐contrast data, may also play a role in the registration error. Interestingly, the whole heart had a significantly higher Dice score (0.92–0.95) than both the LV and TA, possibly due to its well circumscribed pericardial border, less volumetric deformation than the LV, larger local thickness compared to the TA (making single pixel mismatches less important), and more globular structure compared to the tubular TA.

Though limited by the small numbers in this pilot study, potentially pertinent negative findings include a lack of difference between any region of interest or metric for intramodal MR registration over time (Group B) versus intramodal MR registration pre/post‐contrast (Group C), suggesting contrast has little effect on MR registration, and only a single metric difference (HD in the TA) between intermodal registrations at the same time (Group D) and intermodal registrations across time (Group E).

While multimodality imaging can improve target/volume localization,^30^ interscan and delineation variability remain an inherent source of error with regards to quantifying and comparing volumes of interest. In the current study, calculated CT volumes were greater than corresponding MR volumes in almost 70% of individual comparisons. Nevertheless, all population‐averaged RVDs (before DIR) were within 2% (Table 2), and the average ratio of CT volumes to MR volumes was only 1.01. Lack of cardiac gating, motion artifacts (including difficulty of breath‐holding in some lung cancer patients), noise, differences in patient positioning, and challenges in delineating boundaries in CT and non‐contrast MR imaging were possible contributors to the small differences in volumes. Similar studies have shown that the contrast protocol, number of slices, inclusion/exclusion of papillary muscles and trabeculation, and using the same CT volumetry method as CMR impact the differences in ventricular volume between CT and CMR.^31^

The results of this registration study, including adherence to AAPM guidelines, increase confidence in using the evaluated non‐cardiac‐gated multimodal imaging techniques (which were selected to match the available clinical imaging for patients undergoing RT) for future clinical applications. Specifically, longitudinal evaluation of dose‐dependent RT‐induced damage requires registering MR data onto planning CTs from which quantitative dosimetry maps are formed. Notably, cardiac magnetic resonance (CMR) is an established technique to assess the function and structure of the heart (e.g., ventricular volumes, ejection fraction, left ventricular strains, LGE, and tissue characterization using T1/T2 mapping),^32^, ^33^ particularly for evaluating chemotherapy and radiotherapy‐induced CVT in long‐term thoracic cancer survivors (e.g., lung, breast, or lymphatic tissue).^33^, ^34^, ^35^, ^36^ For example, prior studies have shown that CMR can detect significant T1 changes, declining ejection fraction, abnormalities in myocardial strain, and left atrial‐scar enhanced volume following RT.^37^, ^38^, ^39^, ^40^ Thus, as long as radiation dosing is performed solely on CT, DIR will serve as an essential bridge to map quantitative spatially heterogeneous dosing onto pre‐ and post‐RT MRIs, with the goal of improving the early diagnosis and mitigation of CVT. In addition, multimodal image registration is also being used during target definition for radiotherapy of primary and secondary cardiac malignancies and for radioablation of ventricular tachycardia.^14^, ^41^

A few limitations of this study are noted. First, the results of this study are most applicable to the specific modalities and sequences utilized. Notably, both the CT and MR imaging performed were non‐cardiac‐gated based on the clinical and research protocols under which the images were acquired. Using cardiac‐gated CT imaging and CMR would be expected to provide improved registration and additional information of all cardiovascular structures, including subregions of the heart and ventricles. However, the standard thoracic CT and MR evaluated herein provide larger coverage of the chest, including the aorta, and are more directly aligned when both are acquired in axial slices, as opposed to cardiac aligned imaging planes typical in CMR. In addition, T1 images in this study were acquired with a small slice thickness of up to 2 mm which reduces large registration uncertainties. No other MR techniques were investigated in this initial study, and we note that different levels of registration accuracy are possible when CT images are registered to varying MRI techniques. For example, a recent phantom study showed that DIR accuracy between CT and T2 MRI is superior to registration to T1 MRI using a standard head protocol.^42^ Finally, as a pilot study on DIR before a larger CVT study, the sample sizes are relatively small. We note that the assumption of normality was inspected for all data by assessment of skewness from the normal curve in the quantile plot before any further analyses to increase confidence in any observed significant differences. Future analyses with larger sample sizes will be beneficial to address these limitations.

## 5 CONCLUSION

This study confirmed that the accuracy of intra‐ and inter‐modality DIR of the heart, LV, and TA for longitudinal non‐cardiac‐gated 4D‐CT and pre‐ and post‐contrast MR data at inhalation and exhalation is within the recommended limits by the AAPM TG‐132.^1^ Thus, quantitative analyses of these cardiovascular structures of interest in CT and MR images from patients undergoing radiotherapy may be spatially correlated without significant error following registration, even across modalities. These results will provide insight and confidence for future multimodal longitudinal evaluations of cardiovascular function and dose‐dependent CVT.

## AUTHOR CONTRIBUTIONS

All listed authors contributed to the study and to drafting the manuscript.

## CONFLICT OF INTEREST

None.
